# Lectin complement pathway initiators after subarachnoid hemorrhage — an observational study

**DOI:** 10.1186/s12974-020-01979-y

**Published:** 2020-11-12

**Authors:** Jeppe Sillesen Matzen, Charlotte Loumann Krogh, Julie Lyng Forman, Peter Garred, Kirsten Møller, Søren Bache

**Affiliations:** 1 Department of Neuroanaesthesiology, The Neuroscience Centre, Rigshospitalet, University of Copenhagen, Blegdamsvej 3, 2100 Copenhagen Ø, Denmark; 2 Section of Biostatistics, Department of Public Health, Faculty of Health and Medical Sciences, University of Copenhagen, Copenhagen, Denmark; 3 Laboratory of Molecular Medicine, Department of Clinical Immunology, Section 7631, Rigshospitalet, University of Copenhagen, Copenhagen, Denmark; 4 Department of Clinical Medicine, Faculty of Health and Medical Sciences, University of Copenhagen, Copenhagen, Denmark

**Keywords:** Ficolin, Lectin complement pathway, Subarachnoid hemorrhage, Delayed cerebral ischemia, Functional outcome

## Abstract

**Background:**

This exploratory study investigated the time-course of lectin complement pathway (LCP) initiators in cerebrospinal fluid (CSF) and plasma in patients with subarachnoid hemorrhage (SAH), as well as their relationship to delayed cerebral ischemia (DCI) and functional outcome.

**Methods:**

Concentrations of ficolin-1, ficolin-2, ficolin-3, and mannose-binding lectin (MBL) were analyzed in CSF and plasma from patients with SAH. Samples were collected daily from admission until day 9 (CSF; N__PATIENTS_ = 63, n__SAMPLES_ = 399) and day 8 (plasma; N__PATIENTS_ = 50, n__SAMPLES_ = 358), respectively. Twelve neurologically healthy patients undergoing spinal anesthesia and 12 healthy blood donors served as controls. The development of DCI during hospitalization and functional outcome at 3 months (modified Rankin Scale) were registered for patients.

**Results:**

On admission, CSF levels of all LCP initiators were increased in SAH patients compared with healthy controls. Levels declined gradually over days in patients; however, a biphasic course was observed for ficolin-1. Increased CSF levels of all LCP initiators were associated with a poor functional outcome in univariate analyses. This relationship persisted for ficolin-1 and MBL in multivariate analysis after adjustments for confounders (age, sex, clinical severity, distribution and amount of blood on CT-imaging) and multiple testing (1.87 ng/mL higher in average, 95% CI, 1.17 to 2.99 and 1.69 ng/mL higher in average, 95% CI, 1.09 to 2.63, respectively). In patients who developed DCI compared with those without DCI, CSF levels of ficolin-1 and MBL tended to increase slightly more over time (p_interaction = 0.021 and 0.033, respectively); however, no association was found after adjustments for confounders and multiple testing (p-adj_interaction = 0.086 and 0.098, respectively). Plasma ficolin-1 and ficolin-3 were lower in SAH patients compared with healthy controls on all days. DCI and functional outcome were not associated with LCP initiator levels in plasma.

**Conclusion:**

Patients with SAH displayed elevated CSF levels of ficolin-1, ficolin-2, ficolin-3, and MBL. Increased CSF levels of ficolin-1 and MBL were associated with a poor functional outcome.

**Trial registration:**

This study was a retrospective analysis of samples, which had been prospectively sampled and stored in a biobank. Registered at clinicaltrials.gov (NCT01791257, February 13, 2013, and NCT02320539, December 19, 2014).

**Supplementary Information:**

The online version contains supplementary material available at 10.1186/s12974-020-01979-y.

## Background

Aneurismal subarachnoid hemorrhage (SAH) is a life-threatening condition accounting for ~5% of all stroke cases. Despite low annual incidence rates [1], the total loss of productive life years in the SAH patients is similar to more common types of stroke [2], due to a high mortality rate [1] and a low chance that survivors return to the labor market [2, 3].

Delayed cerebral ischemia (DCI) is a main contributor to a poor functional outcome in patients surviving SAH [4, 5]. The pathogenesis and pathophysiology of DCI are still incompletely understood. Previously, DCI has been attributed to cerebral vasospasm [6], but recent research suggests a multifactorial origin, including neuroinflammation as a central causal factor [7, 8].

The complement system is part of the innate immune system, which comprises a first-line defense against invading microorganisms, enabling clearance of dying cells, and amplifying adaptive and inflammatory immune responses upon stimulation [9]. Three pathways activate the complement system, i.e., the classical, the alternative, and the lectin complement pathway (LCP) [10]. The initiators of the LCP, comprising ficolin-1, ficolin-2, and ficolin-3, mannose-binding lectin (MBL), and collectin-10 and collectin-11, induce down-streams activation upon binding to specific carbohydrate structures on injured cells and microorganisms [11, 12].

Recent studies have reported an association between LCP initiators in plasma and functional outcome in ischemic stroke patients [13–16]. LCP initiators have previously been investigated in SAH patients but with conflicting results [17–20].

We aimed to investigate the time-course of LCP initiator levels in CSF and plasma after SAH and to describe their potential relationship to DCI and functional outcome.

## Methods

### Standard protocol approvals, registrations, and patient consents

This study was a retrospective analysis of samples from CSF and plasma, which had been prospectively sampled and stored in a biobank, and some of which were included in a prospective observational cohort study of microRNAs [21]. Protocols were approved by the Danish Scientific Ethics Committee of the Capital Region (H-3-2013-009 and H-3-2014-073) including amendments for the present study (H-3-2013-009 and H-6-2014-073) and the Danish Data Protection Agency (I-suite # 02116 and 03279) and registered at clinicaltrials.gov (NCT01791257 and NCT02320539). Informed consent was obtained from the next of kin, the general practitioner and, when possible, from the patient.

This manuscript adheres to requirements set by the STrengthening the Reporting of OBservational studies in Epidemiology (STROBE) statement.

### Study participants

Patients with spontaneous aneurysmal subarachnoid hemorrhage (SAH) as diagnosed by computed tomography (CT) and CT angiography who were admitted to the Neurointensive Care Unit at Rigshospitalet (Copenhagen, Denmark) were assessed for eligibility. Inclusion criteria were >18 years of age. Exclusion criteria were more than 24 h uncertainty of the time of ictus, as well as the inability to speak and understand Danish.

Neurologically healthy patients >18 years of age with an American Society of Anesthesiologists physical status classification ≤2, who had spinal anesthesia before lower limb orthopedic surgery at Frederiksberg Hospital, were included and served as the control group for the CSF analyses.

Neurologically healthy blood donors >18 years of age were included from the National Donor Registry (Copenhagen, Denmark) and served as the control group for the plasma analyses.

### Clinical definitions and outcome measures

The clinical severity of SAH was assessed using the World Federation of Neurosurgical Societies (WFNS) grading scale, which categorizes patients from 1 to 5; 1 indicating the lowest and 5 indicating the highest disease severity [22]. The extent of intracranial hemorrhage on admission CT was registered by the Fisher grading scale, which rates the severity of SAH from 1 to 4 based on the distribution and amount of blood on CT scans of the head [23].

Clinical deterioration due to DCI was defined according to Vergouwen et al. as the development of a new neurological deficit or a decrease of at least 2 points in GCS, persisting for at least 1 h, and which was not explained by any other potential cause [5]. Two clinicians independently reviewed all patient data obtained in the medical journal within 3 weeks from ictus and allocated the patients to five subgroups regarding the diagnostic certainty of DCI (definite DCI, probably DCI, possible DCI, probably not DCI, and definitely not DCI). To increase the chance of detecting a difference between groups, only patients from the groups, ‘definite DCI’ and ‘definitely not DCI’ were compared.

Functional outcome at 3-months follow-up was based on the modified ranking scale (mRS) [24], which measures the degree of disability (i.e., dependence in daily activities) after a stroke ranging from a mRS score of 0 (no disability symptoms) to 6 (death). This outcome was subsequently dichotomized into good functional outcome, defined as independence or minor disability (mRS 0–2), and poor functional outcome, defined as being dependent or dead (mRS 3–6).

### CSF samples

From patients with an external ventricular drain (EVD), CSF was collected immediately after placement with a sterile procedure, and once daily using an aseptic procedure the following 7 days. From neurologically healthy patients, one CSF sample was collected using a sterile procedure immediately before spinal anesthesia for orthopedic lower limb surgery. CSF samples were immediately put on ice and spun within 15 min at 500 g for 10 min, and the supernatant was stored at −80 °C.

### Plasma samples

Blood was collected from an arterial catheter from SAH patients upon admission and once daily the following 7 days. If no arterial catheter was present, sampling was done from a central venous catheter. From healthy volunteers, blood was sampled once during a blood donation. Blood samples were immediately put on ice and spun within 15 min at 2000 g for 10 min, and the plasma samples were stored at −80 °C. Both plasma and CSF samples were collected at the same time of the morning each day.

### Protein quantification

The concentration of LCP initiators (ficolin-1, ficolin-2, ficolin-3, and MBL) in CSF and plasma was determined using sandwich enzyme-linked immunosorbent assay (ELISA) developed in the Laboratory of Molecular Medicine, Rigshospitalet, according to previously described procedures [25–28]. Briefly, microtiter plates (Nunc Immuno Plates, F384 Maxisorp) were coated with one of the following monoclonal antibodies against ficolin-1 (HP9039; Hycult Biotech, Uden, Nederlands) and in-house produced biotinylated antibodies against ficolin-2 (anti-ficolin 2 ficolin-219), ficolin-3 (anti-ficolin 3 ficolin-334), and MBL (anti-MBL Hyb-131-1) and diluted in phosphate-buffered saline +0.05% Tween-20 (PBS-T), and incubated overnight. Samples were diluted 1:200 and run in triplicates and parallel to a standard pool with a known concentration. Subsequently, polyclonal donkey anti-rapid antibody (ficolin-1) or streptavidin-horseradish peroxidase conjugate (ficolin-2, ficolin-3, and MBL) was applied and plates were developed using o-phenylenediamine dihydrochloride substrate and stopped by adding H_2_SO_4_. Assays were optimized for automated analysis in a 384-well format using the Biomek FX platform (Beckman Coulter, Fullerton, California, USA) for pipetting followed by a DTX880 Multimode detector system where assays were analyzed at 490 nm.

The lower detection limit was as follows in plasma: 5 ng/ml for ficolin-1, 0.005 μg/ml (5 ng/ml) for ficolin-2, 1 ng/ml for ficolin-3, and 20 ng/ml for MBL; it was 0.4 ng/ml in CSF for all four biomarkers. We obtained the following coefficients of variation as calculated from the raw triplicate measurements: Ficolin-1: CSF 9.3%, plasma 7.1%; ficolin-2: CSF 20.2%, plasma 10.6%; ficolin-3: CSF 18.1%, plasma 9.6%, MBL: CSF 9.2%, plasma 7.7%.

### Statistical analysis

Plasma and CSF levels of LCP initiators on admission and specific days after the initial bleeding in SAH patients were compared with those of neurologically healthy individuals using Wilcoxon’s rank-sum test. Within SAH patients, levels were compared between the groups, ‘definitely DCI’ vs ‘definitely not DCI’ and ‘poor functional outcome’ vs ‘good functional outcome’, using a linear mixed model with subgroup (DCI or functional outcome) and day as fixed effects, and patients as a random effect. Crude comparison was done without including any further covariates (univariate analyses), whereas adjusted analyses included sex, age, and WFNS and Fisher scores as covariates (multivariate analyses). The linear mixed model was applied twice: (1) Assuming no interaction (i.e., assuming that LCP initiator levels did not develop differently over time between groups) thereby evaluating the overall difference in concentrations (p), and (2) including the group*day interaction to explore potentially different time developments between groups (p_interaction). Goodness of fit was assessed by residual plots. Due to substantial skewness, CSF levels were log-transformed prior to analysis and results were expressed accordingly (i.e., as percentwise differences in medians). *P* values were corrected for multiple testing using the Benjamini-Hochberg procedure.

All statistical analysis was performed using Statistical Analysis System v.9.4 (SAS Institute Inc., Cary, NC).

## Results

In total, 82 patients were recruited for this study and included in the CSF study alone (*N* = 32), the plasma study alone (*N* = 19), or both (*N* = 31) (Table 1). The 63 SAH patients in the CSF study all underwent placement of an EVD for clinical reasons and had a total of 402 CSF samples collected (Sup. Fig. 1), of which 399 were used for final analysis. Of these 63 patients, 39 (62%) were in a poor condition on admission (WFNS grade 4 or 5) and 13 (21%) had a Fisher grade of 4 (i.e., intraventricular hemorrhage or intracerebral hemorrhage). Thirty-six were assessable for development of DCI; of these, 18 were categorized as ‘definite’ and 18 as ‘definitely not DCI’. Clinical deterioration due to DCI typically occurred between day 6 and day 7 (mean = 6.5 days, SD = 1.5). At the 3-month follow-up, all 63 SAH patients were assessable for a functional outcome, of whom 29 (46%) had a poor (mRS 3–6) and 34 (54%) had a good functional outcome (mRS 0–2), respectively. Twelve CSF samples were collected from 12 neurologically healthy patients.

**Table 1:** Demographic data of the SAH patients and related control groups

	CSF control group (*n* = 12)	CSF (*n* = 63)	Plasma Control Group (*n* = 12)	Plasma (*n* = 50)
**Age**, mean years (SD)	69 (± 9)	59 (±11)	55 (±7)	61 (± 11)
**Sex**, *n* (%)				
Men	8 (66.7)	14 (22.2)	4 (33.3)	5 (10)
Women	4 (33.3)	49 (77.8)	8 (66,7)	45 (90)
**WFNS**, *n* (%)				
1		8 (12.7)		17 (34.0)
2		12 (19.0)		8 (16.0)
3		4 (6.3)		5 (10.0)
4		21 (33.3)		10 (20.0)
5		18 (28.6)		10 (20.0)
**Fisher score**, *n* (%)				
1		0 (0)		0 (0)
2		1 (1.6)		4 (8.0)
3		48 (77.4)		37 (74.0)
4		13 (21.0)		9 (18.0)
**Definite DCI**, *n* (%)		18 (28.6)		13 (26.0)
**Definitely not DCI**, *n* (%)		18 (28.6)		20 (40.0)
**Days from ictus to DCI**, mean (SD)		6.5 (±1.5)		7.2 (±2.2)
**mRS**, *n* (%)				
0–2		34 (54.0)		35 (70.0)
3–6 (poor functional outcome)		29 (46.0)		15 (30.0)
**Aneurysm location**, *n* (%)				
Anterior cerebral artery		25 (39.7)		16 (32.0)
Middle cerebral artery		13 (20.6)		10 (20.0)
Posterior cerebral artery		16 (25.4)		18 (36.0
Internal carotid artery		9 (14.3)		6 (12.0)
**Technique for securing aneurysm**, *n* (%)				
Coil		42 (66.7)		35 (71.4
Clip		21 (33.3)		14 (28.6)
**Vasospasm**, *n* (%)				
Yes		28 (73.7)		17 (70.8)
No		10 (26.3)		7 (29.2)

*CSF* cerebrospinal fluid, *WFNS* World Federation of Neurological Surgeons scale, *EBI* early brain injury, *DCI* delayed cerebral ischemia, *mRS* Modified ranking scale

From the 50 patients in the plasma study, 359 plasma samples were collected (Sup. Fig. 2), of which 358 were used for final analysis. Of these 50 SAH patients, 20 (40%) were in a clinically poor condition (WFNS 4–5) on admission and 9 (18%) had a Fisher grade of 4 (i.e., intraventricular hemorrhage or intracerebral hemorrhage). Thirty-three SAH patients were assessable for the development of DCI, of which 13 patients were categorized as ‘definite’ and 20 patients as ‘definitely not’ DCI. On average, clinical deterioration due to DCI occurred on day 7 (mean = 7.2, SD = 2.2). At the 3-month follow-up, all 50 SAH patients were assessable for the functional outcome, of whom 15 (30%) had a poor and 35 (70%) had a good functional outcome, respectively. Twelve plasma samples were collected from 12 healthy volunteers.

### LCP initiator concentrations in SAH patients vs healthy controls (Fig. 1)

Ficolin-1, ficolin-2, and MBL were not detected in CSF from neurologically healthy patients and ficolin-3 concentrations in CSF were low compared with SAH patients. Median levels of ficolin-1, ficolin-2, and ficolin-3, and MBL were elevated in CSF on all days after SAH compared with neurologically healthy patients; concentrations were high on admission followed by a gradual decline towards normal levels on day 7 to 9. In contrast, ficolin-1 showed a biphasic slope with a second peak between day 3 and 9.

**Fig. 1:**
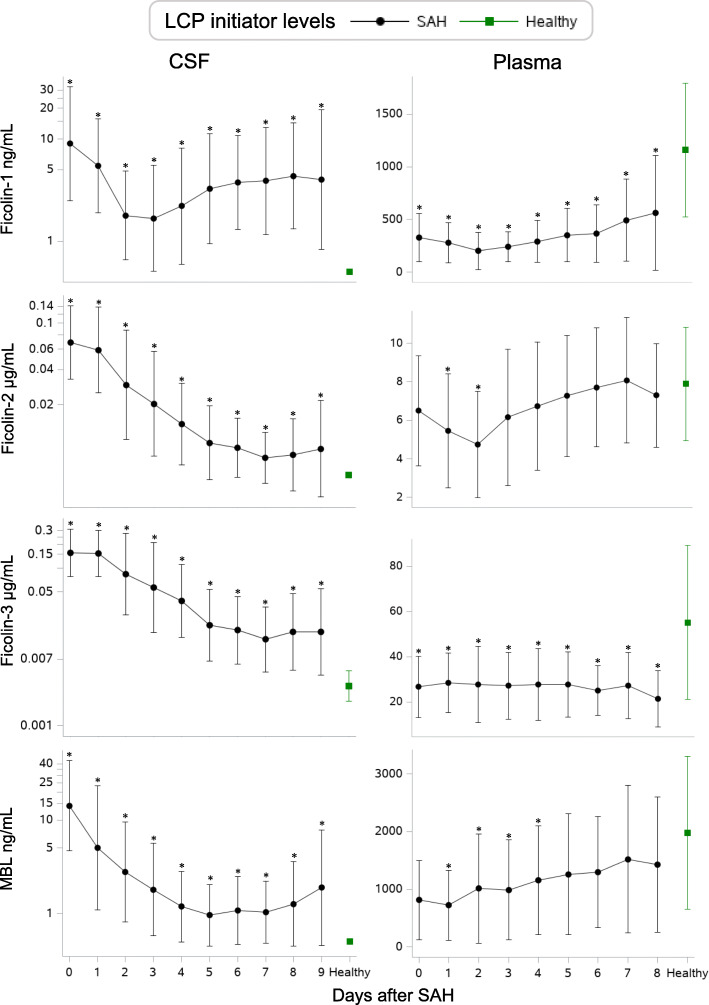
Daily levels of LCP initiators. Daily levels of ficolin-1, ficolin-2, ficolin-3, and MBL in CSF (left) and plasma (right) in patients with SAH (black) and neurologically healthy patients (green). The daily levels are presented as medians in CSF and as means in plasma. Error bars show ±1 SD. Asterisks (*) indicate significance on specific days after SAH compared to control patients (*p* < 0.05) using Wilcoxon’s rank-sum test. Each LCP initiator graph is based on a total number of 399 CSF samples from 63 SAH patients (mean number of samples per patient, 7; range, 6–8), 358 plasma samples from 50 SAH patients (mean number of samples per patient, 7; range, 6–8), and 12 control samples from 12 neurologically healthy patients. MBL: Mannose-binding lectin, CSF: Cerebrospinal fluid, SAH: Subarachnoid hemorrhage

Average plasma concentrations of all LCP initiators in SAH patients were lower than or comparable to the levels of neurologically healthy individuals. Ficolin-1 and ficolin-3 were lower on all days after SAH, whereas ficolin-2 and MBL were lower only on days 1–2 and 1–4, respectively. Ficolin-2 showed a biphasic slope with a second peak from day 2. Ficolin-1 showed a minor decline between day 0 and day 2 followed by a gradual day-by-day increase. MBL showed a gradual increase over time, reaching normal levels from day 5. Ficolin-3 remained low on all days.

Daily levels of the LCP initiators in SAH and neurologically healthy patients are shown in Fig. 1.

### DCI — longitudinal analysis (Fig. 2 and Table 2)

For the 36 patients in the CSF study who were diagnosed as either ‘definitely DCI’ or ‘definitely not DCI’, 241 CSF samples were available.

**Fig. 2:**
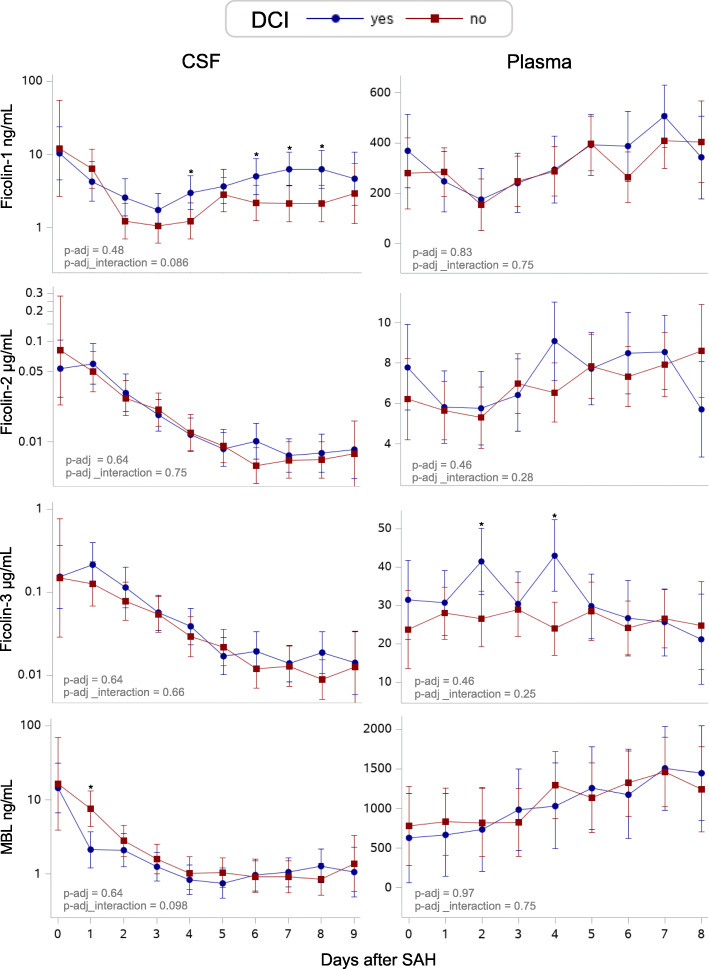
Predicted daily LCP initiator levels in SAH patients with or without the development of DCI. Predicted daily levels of ficolin-1, ficolin-2, ficolin-3, and MBL in CSF (left) and in plasma (right) in patients with (blue) or without (red) the development of DCI. The predicted daily levels are presented as medians in CSF and as means in plasma. Error bars show 95% confidence intervals. *P* values shown in the bottom left of each graph are adjusted for confounders (age, sex, WFNS, and Fisher score) and multiple testing (Benjamini-Hochberg procedure) and represent the overall difference in concentration between groups (p-adj) and overall different development over time (p-adj_interaction) for each LCP initiator. Asterisks indicate (*) significant levels on specific days <0.05. Each LCP initiator graph is based on a total number of 241 CSF samples from 36 SAH patients (mean number of samples per patient, 7; range, 6–8) and 233 plasma samples from 33 SAH patients (mean number of samples per patient, 7; range, 6–8). LCP: Lectin complement pathway, MBL: Mannose-binding lectin, CSF: Cerebrospinal fluid, CT: Computed tomography, DCI: Delayed cerebral ischemia, SAH: Subarachnoid hemorrhage

**Table 2:** Overall difference in CSF levels of each LCP initiator in SAH subgroups (DCI and functional outcome)

	Univariate analyses			Multivariate analyses (age, sex, WFNS, Fisher)		
CSF-levels	Estimate (95% CI)	p	p-adj	Estimate (95% CI)	p	p-adj
**DCI**						
Ficolin-1 (ng/mL)	1.87 (0.99, 3.53)	0.052	0.20	1.72 (0.85, 3.48)	0.12	0.48
Ficolin-2 (μg/mL)	1.08 (0.73, 1.60)	0.69	0.69	1.11 (0.70, 1.78)	0.64	0.64
Ficolin-3 (μg/mL)	1.26 (0.75, 2.13)	0.37	0.61	1.20 (0.65, 2.23)	0.55	0.64
MBL (ng/mL)	1.21 (0.49, 1.39)	0.46	0.61	0.78 (0.42, 1.47)	0.43	0.64
**Functional outcome**						
Ficolin-1 (ng/mL)	**1.85 (1.12, 3.05)**	0.017*	0.022*	**1.87 (1.17, 2.99)**	0.010*	0.040*
Ficolin-2 (μg/mL)	**1.47 (1.08, 2.01)**	0.015*	0.022*	1.33 (0.96, 1,83)	0.079	0.10
Ficolin-3 (μg/mL)	**1.54 (1.01, 2.36)**	0.043*	0.043*	1.45 (0.92, 2.27)	0.10	0.10
MBL (ng/mL)	**1.69 (1.13, 2.52)**	0.012*	0.022*	**1.69 (1.09, 2.63)**	0.020*	0.040*

Estimates represent the relative overall difference in CSF concentration (relative ∆medians) of each LCP initiator in patients with DCI versus without DCI (reference) and in patients with a poor versus good (reference) functional outcome, respectively. Both univariate linear mixed model analyses (left) and multivariate analyses (right) with adjustment for confounders are shown in the table.

*P* values (*p*) represent the overall difference in CSF concentrations between groups (assuming no interaction). p-adj represent *p* values corrected for multiple testing using Benjamini-Hochberg’s procedure. Asterisks indicate significance levels <0.05.

**∆**: Difference; *CI* confidence interval, *CSF* cerebrospinal fluid, Fisher: extent and location of the intracranial hemorrhage, *LCP* lectin pathway initiators, *MBL* mannose-binding lectin; WFNS: clinical severity on admission

In univariate analysis, the CSF level of ficolin-1 seemed elevated in patients with DCI versus patients without DCI (1.87 ng/mL higher in average, 95% confidence interval [CI], 0.99 to 3.53, *p* = 0.052), although insignificant. However, after adjustment for multiple testing, the association was no longer found (p-adj = 0.20). In multivariate analysis adjusted for sex, age, WFNS, and Fisher score no association was found.

Ficolin-1 and MBL increased slightly more over time in patients with DCI versus without DCI, i.e., a different slope was present (p_interaction = 0.021 and 0.033, respectively; data not shown). Adjusted for confounders (sex, age, WFNS, and Fisher score), the increase over time for ficolin-1 and MBL remained significant, but with further adjustment for multiple testing, the increase over time became non-significant (p-adj_interaction = 0.086 and 0.098, respectively, Fig. 2).

Ficolin-2 and ficolin-3 in CSF did not differ between patients with and without DCI.

For the 33 patients who were diagnosed with either ‘definitely DCI’ or ‘definitely not DCI’, 233 plasma samples were available. Neither overall difference in plasma concentrations (Sup. Table 1) nor overall changes over time was observed for any of the LCP initiators (ficolin-1, ficolin-2, ficolin-3, and MBL) in patients with or without DCI (Fig. 2).

### Functional outcome — longitudinal analysis (Fig. 3 and Table 2)

All 399 CSF samples and all 358 plasma samples were entered into these analyses.

**Fig. 3:**
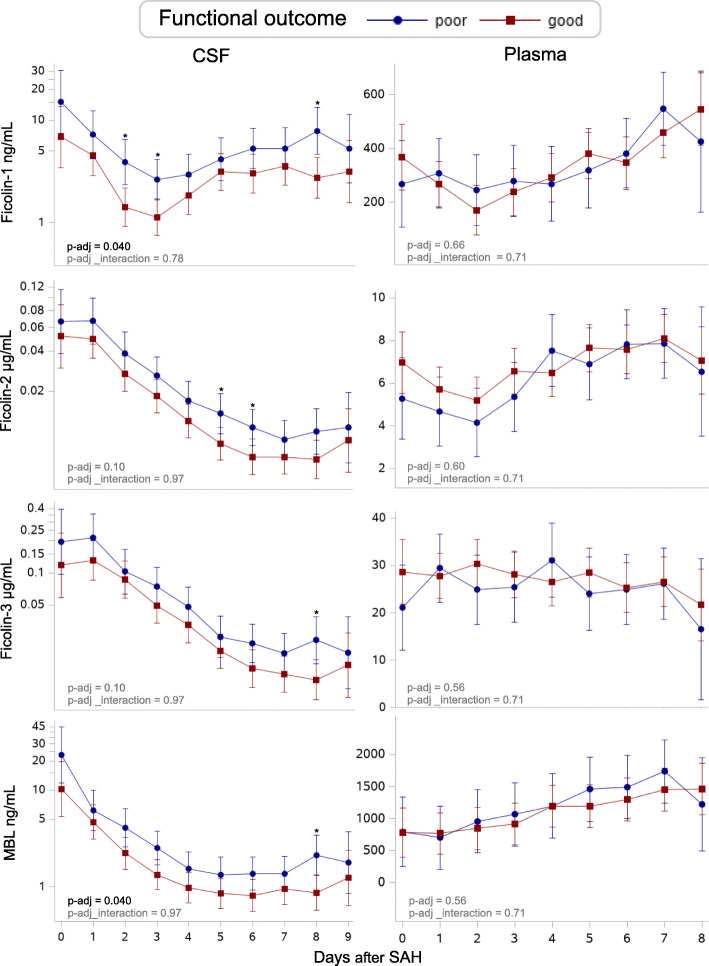
Predicted daily levels of LCP initiators in SAH patients with poor versus good functional outcome. Predicted daily levels of ficolin-1, ficolin-2, ficolin-3, and MBL in CSF (left) and in plasma (right) in patients with a poor (blue) and good (red) functional outcome. The predicted daily levels are presented as medians in CSF and as means in plasma. Error bars show 95% confidence intervals. *P* values shown in the bottom left of each graph are adjusted for confounders (age, sex, WFNS, and Fisher score) and multiple testing (Benjamini-Hochberg procedure) and represent the overall difference in concentration between groups (p-adj) and overall different development over time (p-adj_interaction) for each LCP initiator. Asterisks (*) indicate significant levels on specific days <0.05. Each LCP initiator graph is based on a total number of 399 CSF samples from 63 SAH patients (mean number of samples per patient, 7; range, 6–8) and 358 plasma samples from 50 SAH patients (mean number of samples per patient, 7; range, 6–8). MBL: Mannan-binding lectin, CSF: Cerebrospinal fluid, SAH = subarachnoid hemorrhage

In univariate analyses, CSF levels of ficolin-1, ficolin-2, ficolin-3, and MBL were higher in patients with a poor functional outcome compared with those with a good functional outcome. In multivariate analyses adjusting for sex, age, WFNS, and Fisher score as well as for multiple testing, ficolin-1 and MBL levels remained higher in patients with a poor functional outcome (1.87 ng/mL higher in average, 95% CI, 1.17 to 2.99, *p* = 0.040) and 1.69 ng/mL higher in average, 95% CI, 1.09 to 2.63, *p* = 0.040).

No change over time was detected between groups, indicating that the slopes were parallel (p_interaction >0.05, data not shown).

In contrast, LCP initiators in plasma did not differ between patients with good and those with poor functional outcomes either overall or by time (Fig. 3 and Sup. Table 1).

## Discussion

We investigated the day-by-day changes of LCP initiators in both CSF and plasma after SAH. Our study revealed three major findings. First, CSF concentrations of LCP initiators were increased throughout the first week following SAH, followed by a gradual decline over time. Second, increased CSF levels of all investigated LCP initiators, especially ficolin-1 and MBL, were associated with a poor functional outcome in SAH patients. Third, in univariate analysis, patients with DCI compared with those without DCI, no association was found between DCI and overall LCP initiator levels in CSF. However, ficolin-1 tended to increase slightly more over time in patients with DCI than without (i.e., slopes showing CSF levels increased more over time in the DCI group versus the group without DCI), but the increase was not significant after adjustment for multiple testing (p-adj_interaction = 0.086, Fig. 2).

The present design does not allow us to conclude whether the increased CSF concentration of LCP initiators in the present study is due to local synthesis or simply to a spill-over from the systemic circulation, e.g., due to blood-brain barrier (BBB) dysfunction after SAH. In favor of spill-over from plasma, invasion of complement and inflammatory cells over the disrupted BBB has been proposed as a major contributor to the BBB pathology in other neuroinflammatory central nervous system (CNS) disorders, such as traumatic brain injury, bacterial meningitis, and cerebral malaria [29]. However, this suggestion was based on experimental findings rather than clinical data. Our reported median CSF level of MBL of 15 ng/ml is slightly lower than the median level of approximately 25 ng/ml in patients with pneumococcal meningitis and no MBL deficiency reported in a study evaluating CSF levels of MBL in relation to pneumococcal meningitis, but slightly higher than in such patients with MBL deficiency (<5–15 ng/ml depending on genotype) [30]. Furthermore, another study reported a mean CSF MBL level of 5.11 ng/ml in patients with non-HIV-associated cryptococcal meningitis [31]. To our knowledge, nobody has reported CSF levels of ficolins in pneumococcal meningitis, nor CSF levels of MBL or ficolins in patients with cerebral malaria or traumatic brain injury. In favor of local production of complement proteins, production of initiators and down-stream mediators of the classical and the alternative pathway does occur in the healthy brain [32, 33]. Most LCP initiators (ficolin-2, ficolin-3, and MBL) are primarily produced by hepatocytes in the liver, after which they enter the systemic circulation, forming complexes with mannose-associated serine proteases (MASPs) [34]. Ficolin-1, in contrast, acts as a secretory protein and is mainly produced by the myeloid cell line. Ficolin-1 is found in secretory granules in neutrophils and monocytes, where it can be exocytosed into extracellular environments upon stimulation [35]. We speculate that ficolin-1 is released by immune cells locally within the brain, whereas the remaining LCP initiators enter the intrathecal space from the blood. This hypothesis is supported by the finding that CSF ficolin-1 increased again after an initial decline. Although a concomitant increase in plasma levels could also have facilitated a larger passive influx, the plasma level of ficolin-2 increased in parallel with ficolin-1 but was not associated with an increase in CSF.

One other study has looked at LCP initiator levels in CSF after SAH [17]. The authors reported a tendency towards lower ficolin-1 levels in CSF after 24 h in 18 SAH patients with angiographic vasospasm but did not find any association with the cerebral ischemic lesions. We were not able to directly compare the predicted concentrations in CSF in our study with the previous one because the outcome variables differed (cerebral ischemic lesions versus DCI). However, the previous study found a considerably higher median concentration of ficolin-1 in CSF from patients with cerebral ischemic lesions (51 ng/mL (30–102)) compared with patients with DCI in our study (4 ng/mL (2–8) on day 1). Notably, this could be explained by a different distribution of patients with intraventricular hemorrhage; as many as 84% had intraventricular hemorrhage (i.e., original Fisher scale with grade 4) compared with 21% in the present study, indicating less contamination by blood in our CSF samples. Additionally, the studies used different protein quantification assays.

A study assessing the involvement of the LCP initiators in contused human brain tissue from patients with head trauma found that the LCP initiators were present inside and outside of brain vessels in all contusions examined [36]. Only ficolin-1 was found in normal brain tissue from control patients. In contrast, we were not able to detect ficolin-1 in CSF from neurologically healthy patients. One explanation to this may be that ficolin-1 acts as a secretory protein in the brain, which is only released into the extracellular compartment in response to inflammatory stimulation.

Another study examining brain samples in patients with acute ischemic stroke showed that certain complement proteins, including MBL, were deposited in ischemic lesions in the brain [37]. The authors suggested that increased complement deposition in the brain combined with decreased expression of complement regulators may be a possible mechanism of tissue damage during ischemia. Our findings of increased levels of LCP initiators in CSF associated with SAH and with poor functional outcome appear to support these findings. In line with this, experimental stroke models have reported that complement proteins mediate inflammation after cerebral ischemic injury and that inhibition of complement activation conferred neuronal protection and reduced stroke volume [38–41]. Our findings of increased levels of ficolin-1 and MBL associated with a poor functional outcome may support this notion; however, we have observed an association only and are unable to shed further light on these hypotheses.

Plasma levels of all investigated LCP initiators were lower or comparable in SAH patients compared with healthy individuals, and ficolin-1 and ficolin-3 were lower in both the hyperacute, acute, and the intermediate phase after SAH. Also, circulating LCP initiators were associated neither to DCI nor to functional outcome.

Few research groups have examined the changes of LCP initiators in plasma after SAH, and conflicting results have been reported [14, 17, 18, 20]. As a common denominator, the focus has primarily been on the hyperacute phase of SAH (i.e., hours after ictus). The studies used different approaches (e.g., regarding time from SAH to sampling, definition of DCI, techniques of protein quantification, etc.), which limits the comparability with our study.

This study is the first to provide information about the day-by-day changes of LCP initiators after SAH. The high number of consecutive samples of both CSF and plasma from each patient yielded a higher resolution than previous studies, conferring improved robustness and precision of the natural course of LCP initiators after SAH.

Furthermore, our study complies with the recent reporting recommendations in cellular and molecular SAH studies using recommended methods of sample acquisition, use of collection tubes, biospecimen processing and storage, inclusion of a neurologically healthy control group for both CSF and plasma, as well as using functional outcome and DCI as outcome measures [42]. This way, we hope to contribute to increased homogeneity across studies, which is essential for future biomarker validation to improve risk prediction and optimize treatment. Despite the retrospective study design, patients were thoroughly evaluated for the occurrence of DCI, as well as for their functional outcome 3 months after SAH.

However, this study also has noteworthy limitations. First, we evaluated multiple outcome measures (ficolin-1, ficolin-2, ficolin-3, and MBL) which increase the risk of chance findings. We reduced this risk by correcting for multiple testing.

Due to the post hoc nature of this study, we cannot exclude a potential risk of selection bias as well as a risk of analytical variation due to the chosen method to detect LCP initiators. Furthermore, the retrospective design of the study rendered us unable to measure the actual activity of the LCP system and establish any potential causal relationship with the levels of LCP initiators. However, another study found plasma ficolin-3 and ficolin-3-mediated functional LCP activity to be well correlated [19]. Similarly, a causal relationship between LCP initiators and brain damage is not possible to determine in our study. Experimental studies are needed to address this aspect.

CSF samples were collected from patients treated with an EVD; thus, our CSF findings mainly is related to this selected subgroup of SAH patients. In addition, SAH patients with intraventricular blood (Fisher grade of IV) could conceivably experience variation in the CSF composition depending on positioning and other manipulations affecting cerebrospinal fluid compartments.

All included patients were treated with tranexamic acid (TXA) (an antifibrinolytic) based on the Danish National Guidelines, which might have an impact on complement activity. Many fibrinolytic compounds work as natural C3 and C5 convertases, thus generating C3a and C5a (active anaphylatoxins) [43]. The use of TXA in SAH patients could potentially influence the LCP initiator levels as well as the activity of the complement system.

In our study, we did not control for the presence of intercurrent infection; neither did we measure other markers of inflammation in plasma or CSF. Although SAH is associated with a marked systemic inflammatory response [44, 45], infection, such as pneumonia in mechanically ventilated patients, could also contribute to inflammation and potentially affect the relationship between plasma LCP initiator levels, DCI, and functional outcome. By contrast, the CSF levels of LCP initiators probably represented the true inflammatory response to SAH in and of itself since ventriculostomy-related infection is rare during the first week after insertion [46].

Additionally, a longitudinal analytical approach results in a high-resolution due to frequent repeated measures, which eases the process of visualizing on which time point (day) potential differences between groups occur. However, this analytical approach limits the likelihood of finding significant differences between groups on a specific day. Thus, in our study *p* values for individual days are less important than the visualization of the dynamic changes over time.

Finally, this is still a novel investigation field in which our exploratory and retrospective study design was not able to include all potential laboratory and clinical confounders, which may alter LCP profiles in plasma and CSF. Future studies should investigate comorbidities, in particular on-going inflammatory/infectious diseases, neoplastic, paraneoplastic and demyelinating conditions, premorbid hypertension as stated in recent recommendations as well as other potential confounders [42, 47].

## Conclusion

Our study revealed that SAH patients treated with external ventricular drain had elevated levels of LCP initiators in CSF compared with neurologically healthy patients. Intrathecal ficolin-1 and MBL were increased in SAH patients with a poor functional outcome. No association was found between DCI and overall CSF levels of LCP initiators. Future studies should clarify the pathophysiological mechanism behind ficolin-1 in the central nervous system and determine the potential of ficolin-1 as a prognostic marker of functional outcome.

## Supplementary Information

**Additional file 1.** Supplementary Table 1

**Additional file 2.** Supplementary Fig. 1

**Additional file 3.** Supplementary Fig. 2

## Data Availability

The datasets generated and analyzed during the current study are available from the authors on reasonable request and provided a Data Sharing Agreement approved by the Danish Data Protection Agency.

## Abbreviations

BBB: Blood-brain barrier
CNS: Central nervous system
CSF: Cerebrospinal fluid
CT: Computed tomography
DCI: Delayed cerebral ischemia
EBI: Early brain injury
ELISA: Enzyme-linked immunosorbent assay
EVD: External ventricular drain
LCP: Lectin complement pathway
MASP: Mannose-binding lectin associated serine protease
MBL: Mannose-binding lectin
mRS: Modified ranking scale
SAH: Subarachnoid hemorrhage
WFNS: World Federation of Neurosurgical Societies
