# Supplementary material for: Lectin complement pathway initiators after subarachnoid hemorrhage — an observational study
Source: J Neuroinflammation. 2020 Nov 12;17:338. doi: 10.1186/s12974-020-01979-y (PMC7661172; doi:10.1186/s12974-020-01979-y)
Supplement: Supplementary file 1 — Additional file 1. Supplementary Table 1 [file 12974_2020_1979_MOESM1_ESM.docx]

**Additional file 1: Supplementary Table 1:**

Overall difference in plasma levels of each LCP initiator in SAH subgroups (DCI and functional outcome)


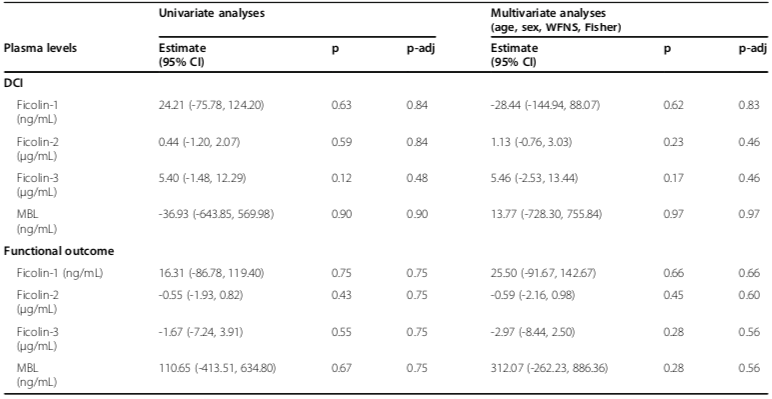


Estimates represents the relative overall difference in plasma concentration (relative ∆means) of each LCP initiator in patients with DCI versus without DCI (reference) and in patients with a poor versus good (reference) functional outcome, respectively. Both univariate linear mixed model analyses *(left)* and multivariate analyses *(right)* with adjustment for confounders are shown in the table.

*P*-values (p) represent the overall difference in CSF concentrations between groups (assuming no interaction). p-adj represent *p*-values corrected for multiple testing using Benjamini-Hochberg’s procedure. Asterisks indicate significance levels < 0.05.

∆: Difference; *CI*: Confidence interval; *CSF*: Cerebrospinal fluid; Fisher: extent and location of the intracranial hemorrhage; *LCP*: Lectin pathway initiators; *MBL*: Mannose-binding lectin; WFNS: clinical severity on admission.
