# Supplementary material for: Lectin complement pathway initiators after subarachnoid hemorrhage — an observational study
Source: J Neuroinflammation. 2020 Nov 12;17:338. doi: 10.1186/s12974-020-01979-y (PMC7661172; doi:10.1186/s12974-020-01979-y)
Supplement: Supplementary file 2 — Additional file 2. Supplementary Fig. 1 [file 12974_2020_1979_MOESM2_ESM.docx]

**Additional file 2: Supplementary Fig. 1**
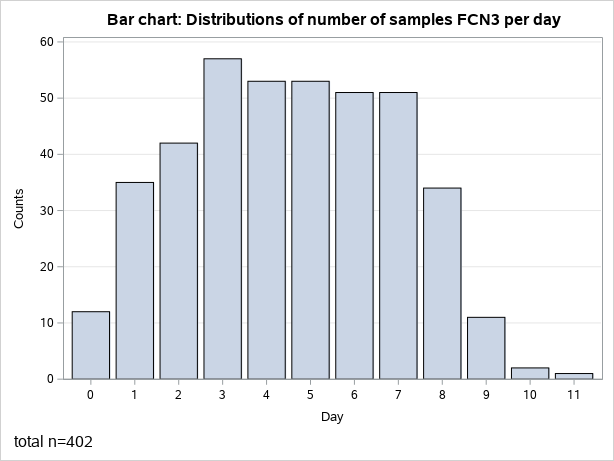


Distribution of CSF samples per day: The figure shows the total number of CSF samples per day. From 63 patients a total number of 402 CSF samples were collected (mean number of samples per patient, 7; range, 6-8).
